# Labelling of ^90^Y- and ^177^Lu-DOTA-Bioconjugates for Targeted Radionuclide Therapy: A Comparison among Manual, Semiautomated, and Fully Automated Synthesis

**DOI:** 10.1155/2017/8160134

**Published:** 2017-05-25

**Authors:** Michele Iori, Pier C. Capponi, Sara Rubagotti, Luca Rosario Esposizione, Johanna Seemann, Riccardo Pitzschler, Thorsten Dreger, Debora Formisano, Elisa Grassi, Federica Fioroni, Annibale Versari, Mattia Asti

**Affiliations:** ^1^Nuclear Medicine Unit, Oncology and Advanced Technologies Department, Arcispedale Santa Maria Nuova-IRCCS, 42123 Reggio Emilia, Italy; ^2^Eckert & Ziegler Eurotope GmbH, Robert-Roessle-Strasse 10, 13125 Berlin, Germany; ^3^Department of Infrastructure Research and Statistics, Arcispedale Santa Maria Nuova-IRCCS, Reggio Emilia, Italy; ^4^Health Physic Unit, Oncology and Advanced Technologies Department, Arcispedale Santa Maria Nuova-IRCCS, 42123 Reggio Emilia, Italy

## Abstract

In spite of the hazard due to the radiation exposure, preparation of ^90^Y- and ^177^Lu-labelled radiopharmaceuticals is still mainly performed using manual procedures. In the present study the performance of a commercial automatic synthesizer based on disposable cassettes for the labelling of ^177^Lu- and ^90^Y-DOTA-conjugated biomolecules (namely, DOTATOC and PSMA-617) was evaluated and compared to a manual and a semiautomated approach. The dose exposure of the operators was evaluated as well. More than 300 clinical preparations of both ^90^Y- and ^177^Lu-labelled radiopharmaceuticals have been performed using the three different methods. The mean radiochemical yields for ^90^Y-DOTATOC were 96.2 ± 4.9%, 90.3 ± 5.6%, and 82.0 ± 8.4%, while for ^177^Lu-DOTATOC they were 98.3%  ± 0.6, 90.8%  ± 8.3, and 83.1 ± 5.7% when manual, semiautomated, and automated approaches were used, respectively. The mean doses on the whole hands for yttrium-90 preparations were 0.15 ± 0.4 mSv/GBq, 0.04 ± 0.1 mSv/GBq, and 0.11 ± 0.3 mSv/GBq for manual, semiautomated, and automated synthesis, respectively, and for lutetium-177 preparations, they were 0.02 ± 0.008 mSv/GBq, 0.01 ± 0.03 mSv/GBq, and 0.01 ± 0.02 mSv/GBq, respectively. In conclusion, the automated approach guaranteed reliable and reproducible preparations of pharmaceutical grade therapeutic radiopharmaceuticals in a decent RCY. The radiation exposure of the operators remained comparable to the manual approach mainly due to the fact that a dedicated shielding was still not available for the system.

## 1. Introduction

Recently, a great interest in the therapeutic applications of radiolabelled analogues of biomolecules targeting specific tumours has been shown both in nuclear medicine research and in clinical practice [1]. Thanks to their smaller dimensions compared to other biological molecules, short chain peptides have been extensively studied as they exhibit favourable pharmacological properties over antibodies or other bioconjugates such as fast tissue penetration, rapid clearance, high target accessibility, and low antigenicity [2]. Thanks to these facts, the feasibility of the imaging process and of the therapy of a large variety of tumours showing receptors specific for the peptide structures has been attested [3]. Nowadays, the most interesting results have been obtained with tumours overexpressing somatostatin receptors by* i.v.* injection of [DOTA]^0^-Tyr^3^-Octreotide (DOTATOC) and [DOTA^0^]-Tyr^3^-octreotate (DOTATATE) (or equivalent analogues) labelled with gallium-68, yttrium-90, and lutetium-177. These radiopharmaceuticals rapidly accumulate in neoplastic tissues allowing the delivery of a high dose of radiation in the target and mainly sparing the surrounding healthy tissues. By this approach it is possible to combine both the diagnosis and the therapeutic process in the same molecule simply by exchanging the radionuclide employed for the labelling [4–6]. As of recently, a multicentric, phase III, clinical study is leading toward marketing authorization of the first somatostatin analogue-based radiopharmaceutical (^177^Lu-DOTATATE, namely, LUTATHERA™) for the treatment of gastroenteropancreatic neuroendocrine tumours.

As prostate cancer (PCa) is one of the most frequent causes of cancer-related mortality in western societies [7], even greater expectations are fuelled by studies on prostate-specific membrane antigen (PSMA) inhibitors labelled with diagnostic and therapeutic radionuclides. PSMA is a type II integral membrane glycoprotein, homologue of N-acetyl-L-aspartyl-L-glutamate peptidase I, that was expressed on the human prostatic carcinoma cell line LNCaP [8]. PSMA is also selectively upregulated in 90–100% of PCa lesions and it is overexpressed in metastatic, poorly differentiated, hormone-refractory carcinomas as well as in cancerous bone metastases and lymph nodes [9, 10]. Furthermore, PSMA has the potential for high-dose radiotherapy thanks to its low expression in healthy tissue, minimizing widespread side effects related to radioactivity. Based on these considerations, novel theranostic bioconjugates were designed for the labelling with both gallium-68 and lutetium-177 and a number of clinical studies on feasibility of diagnosis and treatment of prostate cancer with these labelled analogues have been performed so far [8, 11, 12].

The structure of these theranostic vectors mainly implements the 1,4,7,10-tetraazacyclododecane-1,4,7,10-tetraacetic acid (DOTA) chelator connected to the receptor binding motif (i.e., the pharmacophore glutamate-urea-lysine chain in the case of PSMA or the Phe-D-Trp-Lys-Thr sequence in the case of somatostatin receptors) through a proper linker or through the amino acidic chain of the original biological molecule properly modified for enhancing its stability to the proteases. DOTA is a very efficient bifunctional chelator able to form stable complexes with trivalent metal cations such as yttrium-90 and lutetium-177, the most used radionuclides for peptide receptors radionuclide therapy (PRRT), as well as gallium-68, the most common radio-metal used for positron emission tomography (PET) imaging.

Focusing on therapeutic applications, yttrium-90 is a pure *β*-emitter with a physical half-life of 64.1 h. The emitted electrons show an average energy of 935 keV, a maximum energy of 2284 keV, and are able to penetrate surrounding tissue to up to 12 mm. Lutetium-177 is a medium-energy *β*-emitter, with 6.73 days of physical half-time. The emitted electrons (78.6%) show a maximum *β*-energy of 498 keV and penetrate surrounding tissue up to 2 mm. Lutetium-177 also shows two additional *γ*-emissions (11% and 6.4%) of 210 and 113 keV, respectively [13]. Coupled to the proper carrier, it has been shown that both yttrium-90 and lutetium-177 are able to deliver the required dose for the treatment of extended tumour lesions or micrometastases, respectively [14, 15].

However, the preparation of ^90^Y- and ^177^Lu-labelled radiopharmaceuticals is still mainly performed using manual procedures and the radioactivity handled during the synthesis and fractioning of these radiotracer is up to 19 GBq for yttrium-90 and 37 GBq for lutetium-177, respectively. Radiation protection requirements related to manipulations of a large amount of radioactivity have always been considered one of the main limitations of this practice. Furthermore, the hazard due to radiation exposure demands a strict rotation of operators involved in the process and limits the quantity of radioactivity that can be manipulated in a therapeutic session.

In our experience, the introduction of a semiautomatic synthesizer in 2012 for the synthesis and dose fractioning of ^90^Y- and ^177^Lu-radiolabelled peptides led to a performance evaluation and to an optimization of the prototype system [16]. Moreover, a significant decreasing of operators hands dosimetry in comparison with the completely manual process was documented [17, 18]. Only few studies on complete automation of the labelling process of peptides or antibodies with *β*^−^-emitting radionuclides have been reported so far and are limited to certain ligands or radionuclides [19, 20]. Hence, high regulatory demands and significant radiation exposure of the operators make fully automated approaches almost essential. Furthermore, an automated labelling would allow these processes to meet good manufacturing product (GMP) standards [21] and would combine speed and reproducibility of automation with the safety of a remote system.

In the present study the performance of a commercial automatic synthesizer based on disposable cassettes for the labelling of ^177^Lu and ^90^Y-DOTA-conjugated biomolecules (namely, DOTATOC and PSMA-617) was evaluated and compared to a manual and a semiautomated approach. The behaviour of the instrument was evaluated in terms of radiochemical yield (RCY) and radiochemical purity (RCP) of the preparations, radiation exposure to the operators (finger dosimetry), and other practical evaluations such as reliability and reproducibility of the process. Furthermore, applicability of this automated process to a routine clinical procedure of preparation and injection of therapeutic radiopharmaceuticals was tested.

## 2. Materials and Methods

### 2.1. Reagents and Instrumentation

Modular-Lab Eazy automatic synthesizer, disposable cassettes (C0-YDOTAPEP-CM, C0-LUDOTAPEP-CM, C0-YDOTAPSMA-CM, and C0-LUDOTAPSMA-CM), hardware, and reagent kits were supplied by Eckert & Ziegler Eurotope GmbH (Berlin, Germany). The reagents kits included vial 1 (50 mL of isotonic saline solution; 0.9% sodium chloride), vial 2 (50 mL of water for injection), and vial 3 (50 mg of ascorbic acid). The hardware kits were composed by 1x 2 mL Luer Lock sterile syringe, 3x 0.9 × 70 mm injection needle, 3x 1.1 × 30 mm vent needle, 3x 0.6 × 25 mm injection needle, 1x sterile empty 10 mL plastic flat bottom vial (the reaction vial), 2x sterile empty 10 mL glass vials (reagent vial and waste vial), and 1x cation exchange CM cartridge.

DOTATOC and DOTAPSMA-617 were purchased from Gamma Servizi s.r.l. (Pavia, Italy). ^90^YCl_3_ (carrier free) in 0.05 M HCl solution and ^177^LuCl_3_ (>3000 GBq/mg specific activity) in 0.04 M HCl solution were purchased from Perkin-Elmer (Boston, MA, USA) and ITG GmbH (Garching, Germany), respectively. Pharmaceutical grade ascorbic acid and sodium ascorbate were obtained from Farmalabor (Bari, Italy) and metal-free hydrochloric acid 0.1 M, diethylenetriaminepentaacetic acid (DTPA), sodium citrate, and ammonium acetate were purchased from Sigma-Aldrich (Milan, Italy). Disposable injectable water and 0.9% sodium chloride were purchased from B. Braun (Milan, Italy). MilliQ water (resistivity 18.2 MΩ cm) was used for preparing reagent solutions. All reagents were used without further purification and were weighed on a Model BC balance (PBI, Milan, Italy) when necessary.

As part of manual and semiautomated processes, the reaction vial was heated using an Accublock Digital Drybath heater block (Labnet, Woodbridge, New Jersey, USA) with a modified aluminium adaptor. Activity measurements were performed using an Aktivimeter ISOMED 2000 dose calibrator (MED Nuklear-Medizintechnik, Dresden, Germany). Radiochemical purity (RCP) of preparations was assessed by (i) TLC using RP-18F plates (Merck, Whitehouse Station, New Jersey, USA), ITLC-SG plates (Varian, Milan, Italy), and a Cyclone Plus Storage Phosphor system (Perkin-Elmer, Boston, MA, USA) and (ii) HPLC system (ITG, Garching Germany) equipped with an Acclaim 120 C18 column (3 *μ*m, 3 × 150 mm) (Thermo Scientific, Milan, Italy) and a radiochemical and UV detector.

### 2.2. Dose Exposure Evaluation

Operators safety was monitored by ten thermoluminescent fingertip dosimeters (TLD) and one direct reading personal device (DMC 2000 XB), as already described previously [17, 18]. Dosimeter type and personal protection equipment worn by operators remained unchanged in spite of the method used for the labelling.

### 2.3. Manual Labelling

**Figure 1:**
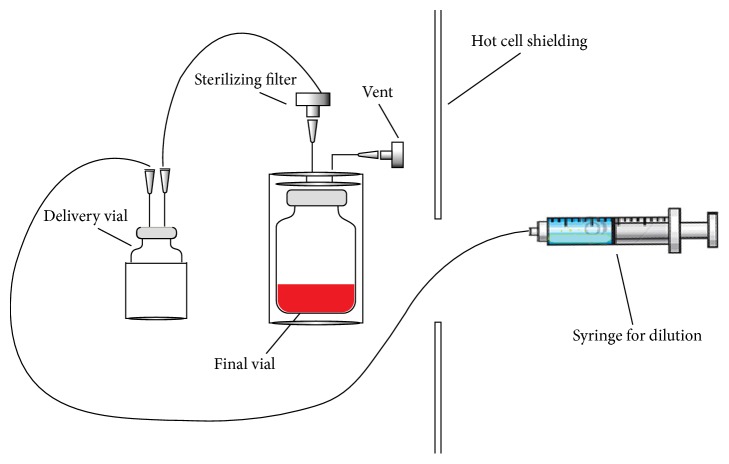
Scheme of the manual assembled system for the filtration and dilution of ^90^Y and ^177^Lu-labelled radiopharmaceuticals.

Manual labelling was carried out using telescopic tongs and dedicated poly(methyl methacrylate) (PMMA) shielding for syringes and vials. Firstly, the delivery vial, containing ^90^YCl_3_ or ^177^LuCl_3_ solutions, was transferred to the proper reaction shielding. A syringe filled with 1 mL of a 0,15 M sodium ascorbate solution (to buffer the reaction to a pH ranging from 4,6 to 4,9) and with a volume of DOTATOC solution proportional to the delivered radioactivity (13 ng/MBq for yttrium-90 and 11 ng/MBq for lutetium-177, resp.) was added to the radionuclide solution. The vial was then transferred to a heating block and heated for 30 min at 90°C. At the end of the reaction, a small aliquot of activity (15–60 MBq) was withdrawn and diluted with 0,3 mL of 0,1 M HCl for assessing RCP. The solution was transferred through a sterilizing filter to a 25 mL vial and diluted in 20 mL of physiological solution by means of a manually assembled system consisting of tubes, valves, and syringes (Figure 1). The final vial already contained a volume 1 mM DTPA solution proportional to the initial activity in order to complex any potentially unlabelled radionuclide. A ZD 100 automatic dispenser (Maspres, Florence, Italy) was used to prepare doses for patients, by fractioning the final bulk in vials containing 2 mL of an ascorbic/ascorbate buffer solution. The ascorbic/ascorbate buffer solution decreases radiolysis phenomena enhancing stability of the products [22].

### 2.4. Semiautomated Labelling

**Figure 2:**
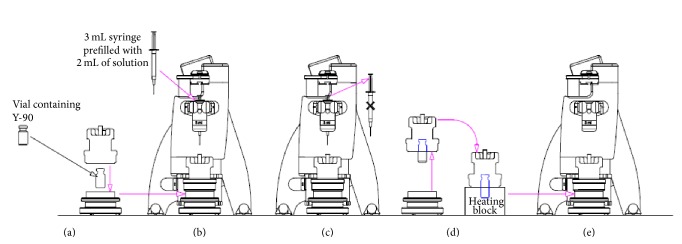
Scheme of operations performed with the ADD-2 semiautomated synthesizer. The delivery vial was placed in the ADD shielding (a), precursor and buffer were added (b), syringe was discharged (c), and vial was placed in the heating block (d) and placed again in the ADD-2 body after heating. The original version of the figure was published in [16].

The radiolabelling was performed by using an ADD-2 (Amercare, Thame, UK), a prototype of a semiautomatic dose dispenser device modified for performing simple radiolabelling procedures avoiding the use of disposable cassettes as already described previously [16]. The system included a special shielding and filter holder to perform sterile filtration of the final product. Radiolabelling was carried out by following the same steps of the manual method, but the operations of injection of the precursor, withdrawing for quality control, dilution, filtration, and fractionation were performed in remote by using the ADD-2 features as shown in Figure 2.

### 2.5. Fully Automated Radiolabelling

**Figure 3:**
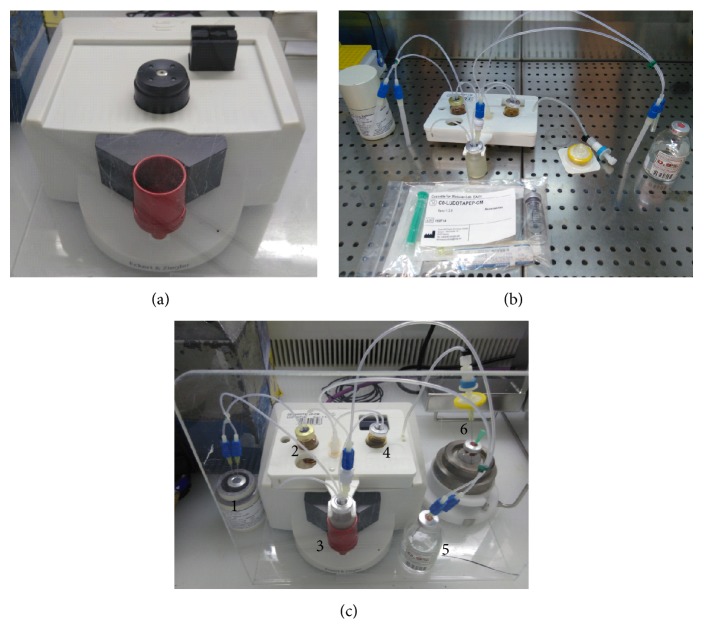
Images of the ML Eazy device (a), a disposable cassette (b), and disposable cassettes assembled onto the system (c). Delivery vial (^90^Y and ^177^Lu-chloride solutions) (1), reagent vial (precursor and buffer solution) (2), reactor vial (3), waste vial (4), 0.9% NaCl solution vial (5), and product vial (6).

**Figure 4:**
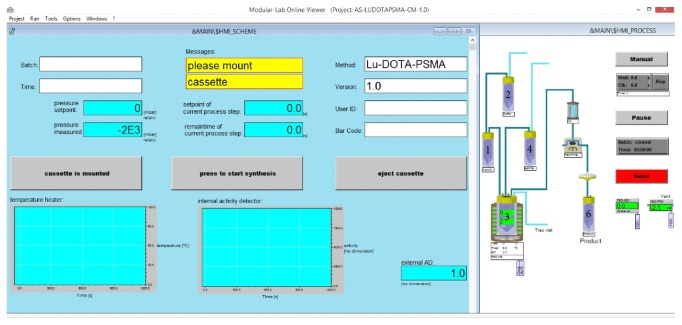
Screenshot of ML Eazy software interface. Synthesis steps, amount of radioactivity in the reactor, and synthesis parameters are displayed during each synthesis run. Delivery vial (^90^Y and ^177^Lu-chloride solutions) (1), reagent vial (precursor and buffer solution) (2), reactor vial (3), waste vial (4) (0.9% NaCl solution vial is missing), and product vial (6).

Radiolabelling was performed by means of a ML Eazy system. The system uses a new technology working with a pressure distribution system instead of stopcocks or solenoid valves for liquids transfer. This internal and independent pressure system is set up inside the body of the synthesizer, while the disposable cassette is mounted on the top of the system simply clicking-in the synthesis cassette (Figure 3). All pressure connections are ensured automatically. After transferring all the required reagents in the proper vials, the cassette is subsequently mounted on the synthesizer. Vial containing the starting activity is connected to the synthesis cassette (delivery vial is kept in lead shielding throughout the whole process) in the last step prior to start the synthesis. The process works without external interaction as all steps are implemented in the synthesis template. The design of the cassettes for the labelling of ^90^Y- and ^177^Lu-PSMA-617 or ^90^Y- and ^177^Lu-DOTATOC is identical allowing easy and intuitive handling and preparation by the operator. Synthesis steps can be monitored via the HMI-scheme (Figure 4). The Modular-Lab software is compliant with GMP standards (annex 11 for computerized systems), 21CFR 210/211 cGMP, GAMP5, and 21CFR part11 regulations.

All consumables and reagents needed (excluding the precursors) were provided as part of the reagent kit. The light CM cartridge, the sterile filter, and a needle were connected to the end of the product line of the cassettes and the needle was inserted through the septum of a 25 mL vented vial. The reagents were prepared and filled as follows: (i) 1.5 mL of water was withdrawn from vial 2 with a syringe and injected into vial 3 containing ascorbic acid. The vial was gently shaken to completely dissolve the substance. (ii) A solution of the precursor (DOTATOC or PSMA-617) proportional to the delivered radioactivity was then added to vial 3. In a first set of experiments, the amount of precursor (DOTATOC or PSMA-617) was kept equal to 40 ng/MBq for both yttrium-90 and lutetium-177 as requested by the specification of the Eazy system supplier (manual version TD047_ETD Rev. 03/12.2014). However, in a second set of experiments, the ratio between DOTATOC and delivered radioactivity was kept equal to that used in the manual and semiautomated approach (i.e., 13 ng/MBq for yttrium-90 and 11 ng/MBq for lutetium-177, resp.) in order to have a better comparison of the results. (iii) The solution from vial 3 was withdrawn with a syringe and slowly injected into the dedicated vented vial of the cassette. Afterwards, the vent needle was discarded.

The cassette was assembled to the Eazy system and vial 1, containing isotonic solution, was connected to the cassette through the dedicated needles. The vial containing yttrium-90 or lutetium-177 chloride solutions was connected to the cassette by inserting the two dedicated needles. The software was started and the project corresponding to the synthesis was selected. After confirming the “preparation steps” displayed the synthesis was started and moved toward the following steps: preheating of reaction vial (90°C), transfer of buffer/precursor solution from buffer vial through delivery vial and then into the reaction vial, reaction time (about 30 minutes of heating), product transfer, and purification and dilution into the product vial through CM and sterile filter. At the end of the synthesis, the cassette was automatically ejected and the residual radioactivity remaining in the principal components (delivery vial, reactor vial, tubing, CM cartridge, and sterile filter) was measured discriminating (i) between DOTATOC or PSMA-617 preparations (*n* = 12) in spite of the radionuclide used (yttrium-90 or lutetium-177) and (ii) between yttrium-90 or lutetium-177 (*n* = 12) in the DOTATOC preparations only. Aliquots for quality control were withdrawn directly from the final vial.

### 2.6. Quality Controls

The RCP of ^177^Lu and ^90^Y-labelled radiopharmaceuticals was assessed (i) by two thin layer chromatography (TLC) systems using ITLC-SG plates and RP-18F plates as the stationary phases. A 1 M ammonium acetate/methanol (1 : 1) solution and a 0.1 M sodium citrate/1 M HCl (97 : 3) solution were used as mobile phases, respectively. Reference standard solutions (radiopharmaceutical, free, and hydrolysed radionuclides solutions) were prepared and analysed as described previously [16] by high performance liquid chromatography (HPLC), injecting 10 *μ*L of the diluted radiopharmaceutical on a Acclaim 120 C18 column and eluting them with the following gradients: A: 0.1% TFA solution solvent; B: acetonitrile; ^177^Lu and ^90^Y-DOTATOC: 0–11 min 82% A, 11–16 min 40% A, and 11–20 min 16% A; ^177^Lu and ^90^Y-DOTA-PSMA: 0–10 min 90% A, 10–13 min 40% A, and 13–16 min 90% A. Absence of bacterial endotoxin and sterility were tested on products according to European Pharmacopeia standards.

### 2.7. Statistical Analysis

Descriptive statistics were performed to investigate sample characteristics; mean and median interval interquartile (IQR) and standard deviation was chosen to summarize continuous variables. The assumption of normality for continuous variables was verified statistically using the Kolmogorov-Smirnov test. The Student* t*-test and the one-way analysis of variance (ANOVA) are used to determine whether there are any statistically significant differences between the means of two or more independent (unrelated) groups. The Kruskal–Wallis test by ranks was used in between-group comparisons for variables not normally distributed. The threshold for statistical significance was set at *p* < 0.05. IBM SPSS Statistics 23 for Windows (SPSS, Chicago, IL) were used for statistical analyses.

## 3. Results

### 3.1. Radiopharmaceuticals Preparation

**Figure 5:**
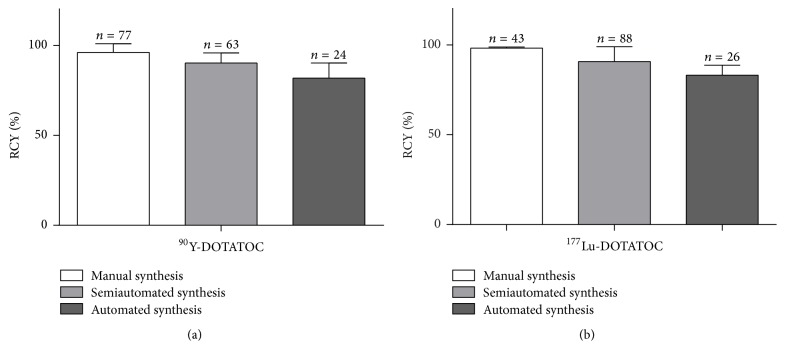
Comparison of the performances of three different approaches preparing ^90^Y- and ^177^Lu-DOTATOC. The precursor/radionuclide ratio was 13 ng/MBq for yttrium-90 and 11 ng/MBq for lutetium-177, respectively.

Between 2007 and 2016, more than 300 clinical preparations of both ^90^Y- and ^177^Lu-DOTATOC have been performed using three different approaches. The labelled radioactivity ranged from 5 to 19 GBq for yttrium-90 and from 16 to 37 GBq for lutetium-177, respectively. The ratio between the peptide amount and the radionuclides radioactivity was kept constant and at least 4 patient doses were prepared from each synthesis. When the manual approach was used, an average radiochemical yield (intended as the ratio between the starting radionuclide radioactivity and the radioactivity of the labelled product in the final vial) of 96.2 ± 4.9% (*n* = 77) and 98.3 ± 0.6% (*n* = 43) for ^90^Y and ^177^Lu-DOTATOC, respectively, was obtained. The synthesis failure rate (intended as preparations where no injectable radiopharmaceutical was achieved) was 0% for both the radionuclides. When the ADD-2 system was used, the average RCYs were 90.3 ± 5.6% (*n* = 63) for ^90^Y-DOTATOC and 90.8 ± 8.3% (*n* = 88) for ^177^Lu-DOTATOC, respectively, with failure rate of 1.6% (1/63) for yttrium-90 and 0% for lutetium-177, respectively. For preparations carried out with the ML Eazy system, average RCYs of 82.0 ± 8.4% (*n* = 24) and 83.1 ± 5.7% (*n* = 26) for ^90^Y- and ^177^Lu-DOTATOC, respectively, were achieved with a failure rate of 4.4% (1/24) for yttrium-90 and 3.8% (1/26) for lutetium-177, respectively. These results are summarized in Figure 5. RCYs of the same radiopharmaceutical (^90^Y- or ^177^Lu- DOTATOC) with the three synthetic approaches were compared through an ANOVA test and the differences were statistically significant.

**Figure 6:**
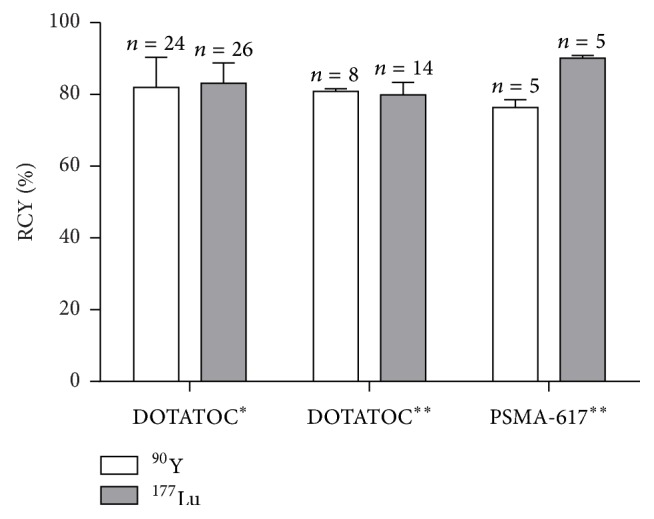
Radiochemical yields obtained with the automated synthesizer ML Eazy with different precursors and concentrations. ^*∗*^13 ng/MBq for yttrium-90 and 11 ng/MBq for lutetium-177 ^*∗∗*^40 ng/MBq for both yttrium-90 and lutetium-177.

Performances of the ML Eazy were studied further by increasing the ratio between radionuclides and precursor up to 40 ng/MBq (as requested by the ML Eazy specifications) obtaining an average RCY of 80.9 ± 0.7% for ^90^Y-DOTATOC (*n* = 8) and 79.9 ± 3.4% for ^177^Lu-DOTATOC (*n* = 14), respectively. The differences in RCY when using the two radionuclide/peptide ratios are both not statistically significant. The ML Eazy system was also tested to prepare ^90^Y- and ^177^Lu-PSMA-617 (not for clinical use; in this case the ratio between radionuclides and precursor was always 40 ng/MBq) and average RCYs of 76.4 ± 2.2% (*n* = 5) and 90.1 ± 0.8% (*n* = 5), for yttrium-90 and lutetium-177, respectively, were achieved. These data are gathered in Figure 6.

The radioactive residues, remaining in parts of the cassettes, are shown in Table 1. The radioactivity was mainly detected in the delivery vial, the reactor, and the CM cartridge. The distribution of the two radiolabelled precursor among the disposable cassette is roughly equivalent except for the CM cartridge where a radioactivity of 6.7% and 0.1% was detected for DOTATOC and PSMA-617, respectively. When the distribution of the two radionuclides was compared, a significant difference in the residual activity of the delivery vial (4.8% for yttrium-90 and 1.1% for lutetium-177) and in the CM cartridge (7.9% for yttrium-90 and 3.6% for lutetium-177) was noted.

### 3.2. Quality Controls

**Figure 7:**
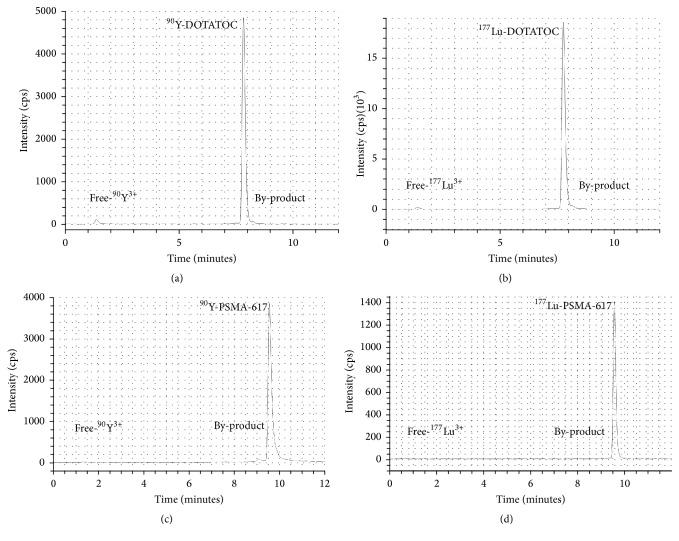
Paradigmatic HPLC chromatograms of radiopharmaceutical preparations: ^90^Y-DOTATOC (a), ^177^Lu-DOTATOC (b), ^90^Y-PSMA-617 (c), and ^177^Lu-PSMA-617 (d).

In spite of the method used, the average RCP of DOTATOC radiopharmaceuticals prepared in this study was generally higher than 99% for TLC analysis and higher than 97% for HPLC analysis and it was independent of both the radionuclides and precursors utilized. HPLC analyses were able to discriminate between free-radionuclide, hydrolysed products and some unknown by-products probably due to radiolysis or oxidation phenomena (Figures 7(a) and 7(b)). Retention times (*R*_*t*_) were as follows: unreacted radionuclides *R*_*t*_ = 1,3–1,4 minutes, ^90^Y/^177^Lu-DOTATOC *R*_*t*_ = 7,7 min, and by-products *R*_*t*_ = 8,2 min. The manual method led to a failure rate (i.e., synthesis not deliverables to patients because the RCP was under the established admissible value of 97%) of 8.0% (6/77) and 7.0% (3/43) for yttrium-90 and lutetium-177, respectively. Conversely, only one synthesis of ^90^Y-DOTATOC prepared both with semiautomated and automated methods was uncompliant and hence not delivered to the patient (failure rates of 1.6% for semiautomated and 4.3% for automated method, resp.). The overall results of both analysis methods are summarized in Table 2. RCP of ^90^Y-/^177^Lu-PSMA-617 preparations was generally higher than 99% by TLC and 98% by HPLC. The retention times were unreacted radionuclides *R*_*t*_ = 1,4 minutes, by-products *R*_*t*_ = 7–9 minutes, and ^90^Y-/^177^Lu-PSMA-617 *R*_*t*_ = 9.5 minutes, respectively. A paradigmatic HPLC chromatogram is shown in Figures 7(c) and 7(d) while overall results of both analysis methods for PSMA-617 are summarized in Table 3.

### 3.3. Dose Exposure

**Figure 8:**
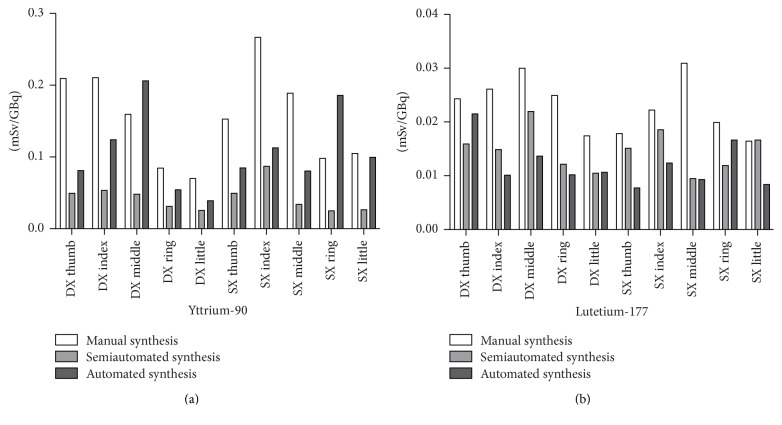
Comparison between the fingertips exposure of the operators during preparation of yttrium-90 (*n* = 77, 63, and 24 for manual, semiautomated and automated approach, resp.) and lutetium-177 (*n* = 43, 88, and 26 for manual, semiautomated and automated approach, resp.) labelled radiopharmaceuticals using three approach. Data are reported as means; SDs are omitted for clarity.

Fingertip exposure data were normalized to the activity used in each procedure. The values obtained with the automatic synthesizer were compared to the values computed from the manual and semiautomated methods. As no practical difference in the operations between the labelling of DOTATOC or PSMA-617 was adopted, the exposure data related to the two radiopharmaceuticals were gathered together. The mean doses on the whole hands for yttrium-90 preparations were 0.15 ± 0.4 mSv/GBq, 0.04 ± 0.1 mSv/GBq, and 0.11 ± 0.3 mSv/GBq for manual, semiautomated, and automated synthesis, respectively. For lutetium-177 preparations, the mean doses were 0.02 ± 0.008 mSv/GBq, 0.01 ± 0.03 mSv/GBq, and 0.01 ± 0.02 mSv/GBq for manual, semiautomated, and automated synthesis, respectively. Differences among the methods are not statistically significant. Detailed results obtained for the single fingers are shown and compared in Figure 8.

Personal equivalent superficial dose [Hp(0.07)] and depth dose [Hp(10)] values did not show significant variations related to labelling methods for both the radionuclides.

## 4. Discussion

The clinical use of therapeutic agents labelled with yttrium-90 or lutetium-177 entails a large exposure to radiation hazards for operators as well as significant costs and a serious investment in the organization of activities. Therefore, an in-depth evaluation of these aspects, together with the necessity to achieve required performances of yield, purity, and reproducibility to satisfy the patients' needs and accomplish the therapeutic program, is of primary importance for the selection of the synthesis method. Furthermore, the encouraging clinical results obtained by labelled biomolecules like somatostatin and PSMA inhibitor analogues [4–7] strongly demands a method capable of managing the labelling of different pharmacophores in a safe, reproducible, and GMP compliant way. The automation of labelling procedures is the logical answer to this challenge. The introduction of a semiautomated system (namely, ADD-2), chemical optimization (pH and buffer type, ligand/radionuclide ratio, and reaction time) of the synthesis, and impact on the radioprotection of operators in the preparation of high doses *β*^−^-emitting radiopharmaceuticals were already described in detail as a part of previous studies [17, 18].

In the present study a 9-year long experience on the preparation of therapeutic radiopharmaceuticals for clinical use (i.e., ^90^Y- and ^177^Lu- DOTA-peptides) was evaluated by evolving through manual, semiautomated, and automated methods. Moreover, the performance of the commercially available automatic synthesizer (ML Eazy) in the preparation of novel promising radiopharmaceuticals such as ^90^Y- and ^177^Lu-PSMA-617 was evaluated.

The manual method assured the highest RCY (>95% for yttrium-90 and >98% for lutetium-177, resp.), thanks to the fact that the reaction occurred directly inside the radioactivity delivery vial. This means that the radionuclides radioactivity is all employed in the labelling and the only source of leakage may happen during transfer and dilution of the labelled precursor to the final vial through a sterile filter. In rare case, when RCY was below 95%, it was due to a defective transfer of the product caused by a leak in the manually assembled tubing system. In spite of this finding, no synthesis failure was observed. The whole procedure took about 120 minutes including preparation of tubing, labelling, and quality controls of the final product. The manual method offered the chance to actively manage the labelling process and stopping or modifying the procedure if something goes in a wrong direction. This opportunity is limited by the main drawback of the method, namely, the high exposure of the operator's hands to radiations. The following handling steps were identified as critical in terms of radiation exposure: (i) manual injection of buffer/ligand solution, (ii) transfer of the original vial from the delivering shielding to the heating block, (iii) transfer from the heating block to the final shielding at the end of the warming, (iv) insertion of the tubes for dilution, and (v) removal of disposables (tubing and sterile filter) at the end of the process. In some of these steps radioactivity was not shielded in a proper PMMA shielding and the only protection for the operator was guaranteed by wearing anti-X 0.20 mmPb-equivalent gloves. In particular, dosimetry was particularly elevated in ^90^Y-DOTATOC preparations due to the high energy *β*^−^-emissions of the radionuclide.

Upon the RCP of the radiopharmaceuticals prepared with this method (Table 2), the multiple manual injections and venting operations increased the probability of air and contaminants to enter the vials, thus enhancing hydrolysis phenomena and partial labelling of the precursors. Moreover, issues during the heating step due to malfunctions of the heating block cannot be excluded. This resulted in a noticeable number of syntheses (6/77 for yttrium-90 and 3/43 for lutetium-177) where RCP of the radiopharmaceutical was not high enough for release. In summary, the labelling performed with manual approach guaranteed high yield but relatively low reproducibility of the process. The cost of the disposable (only tubing, filter, and vial) was almost negligible if compared to the cost of the precursor and the radionuclides.

Similar to the manual approach, the semiautomated methods (ADD-2 system) performed the labelling reaction directly in the delivery vial of the radionuclides. Transferring to and dilution of the radiopharmaceuticals to the final vial were carried out automatically by the system instead. The process took ca. 60 minutes and assured a RCY around 90% for both yttrium-90 and lutetium-177 with the greater loss due to radioactivity remaining in the delivery vial or small leaks during the transfer. These leaks mainly occurred in the last period of use when the original symmetry and perfect overlap of the parts were partially lost. In these cases, a growing laxity of the engine movement and an increase in the software communication failures were also observed. These finding highlighted the greatest drawback of this system, that is, the need of frequent maintenance to maintain a high level of performance. In spite of this, the only synthesis completely failed occurred in a ^90^Y-DOTATOC preparation and was due to a software error that affected communication with the engine. On the other hand, quality controls revealed an excellent incorporation of the radionuclides (>98%, Table 2) and the automation almost extinguished the number of preparations not compliant with the release criteria (1/62 for yttrium-90 and 0/88 for lutetium-177).

If compared to previously described manual method, hands exposure of the operators drastically decreased (meanly 4-fold less for yttrium-90 and 2-fold less for lutetium-177, resp., Figure 8). This is due to the fact that the only operation where the radioactivity was not shielded by the proper ADD-2 shielding was during transfer of the radionuclides vial from its delivery shielding to the ADD-2 one. The preparations involving lutetium-177 attested a greater improvement in dosimetry compared to those involving yttrium-90, as the *γ*-emission and the low-energy *β*^−^-emission of lutetium-177 were more effectively blocked by the thick tungsten ADD-2 shielding while the high energy *β*^−^-particle of yttrium-90 can generate further source of radiations due to Bremsstrahlung phenomena [18]. In summary, the labelling performed with a semiautomated approach resulted in good yields and reproducibility of the process. The radiation exposure of the operator was noticeably improved as well and the cost of the disposables (just vial and syringes) was considered negligible.

The fully automated device (Modular-Lab Eazy) exhibited the advantage of a completely automated process where, after assembling of the disposable cassette and the reagents, the procedure is performed without any interference of the operator. Differently from the two approaches described above, in this method the starting activity is transferred in a disposable reactor where the reaction take place. The mixture is subsequently transferred to the final vial passing a CM cartridge. By using this approach a statistically significant lower RCY (around 83% for both ^90^Y- and ^177^Lu-DOTATOC) was obtained. It is mainly due to the fact that parts of radionuclide solution were not transferred to the reactor and, therefore, remained in the starting vial. Secondly, some radioactivity was lost in the reactor vial and in the CM cartridge (Table 1) during the transfer to the final vial. The results were roughly independent from the amount of precursor used as the same RCYs were obtained when the precursor/radionuclide ratio was increased to 40 ng/MBq. These findings indicate that the labelling reaction occurred precisely and there was no unpredictable increase of competing contaminants in the disposables. RCY was only lowered by radioactive residues lost in the cassette parts. With this method 1 synthesis failure for both ^90^Y- and ^177^Lu-DOTATOC (1/24 and 1/26 run, resp.) was recorded and was due to an omitted transfer of the precursor/buffer solution from the starting vial to the reactor caused by a pressure loss inside the system. The device proved to be able to synthesize ^90^Y- and ^177^Lu-PSMA-617 as well mainly with the same steps utilized for DOTATOC and with a RCY around 76% and 90%, respectively. The good reproducibility of the processes and the presence of the CM cartridge for purifying the labelled products guaranteed an almost certain compliance of the products with RCP always over 97% (Table 2).

In spite of the total automation of the process, the exposure of the operator's hands to radiations was affected by the fact that the ML Eazy synthesizer is not equipped with a dedicated shielding. Because of this, the operator's hands were exposed during connection of the starting vial to the cassettes body, during recovery of the final vial and during removal of the cassettes at the end of the process. As highlighted by the data gathered in Figure 8, all these operations are a source of high exposure mainly due to the high amount of radioactive residues lost in the unshielded disposable parts. For these reasons, the automated synthesizer resulted in a mean hand exposure lower to the manual method but almost 3-fold higher than the semiautomated method when yttrium-90 was handled. The results were more encouraging for lutetium-177 labelling where exposure was almost halved if compared to the manual method and comparable to the semiautomated method. This difference between the two radionuclides is probably due to the fact that the lower energy and shorter range *β*^−^ particles of lutetium-177 could be better absorbed by the anti-x gloves worn by the operator and better reduced by distance. The direct finger to finger comparison of the three methods is summarized in Figure 8. Considering costs of the cassettes and the ready to use GMP reagents, all over spending was higher than the costs of the disposables used with the other methods but it was not significant if compared to the costs of the radionuclides and the ligands considering a high-activity labelling. However, it is worth noticing that the lower RCY compared to the other two approaches is economically impacting because it implies to start the labelling with a radionuclides amount 10–15% higher than the other two methods in order to obtain the same amount of final product to deliver to patients.

In conclusion, the automated approach with the ML Eazy synthesizer guaranteed a decent RCY and a high reliability and reproducibility of the process. The operations are generally simpler and no direct intervention of the operators is needed. The total time needed to prepare the system and perform the labelling was around 45–60 minutes. The radiation exposure of the operator's hands was lowered with respect to the manual approach and could be further improved if a dedicated shielding for the labelling of therapeutic radiopharmaceutical was provided.

## 5. Conclusion

In this study performance of three approaches of different grade of automation for the preparation of ^90^Y- and ^177^Lu-labelled radiopharmaceuticals was compared. The automated approach guaranteed reliable and reproducible preparations of pharmaceutical grade therapeutic radiopharmaceuticals, if potential concerns about the RCY and about the radiation exposure of the operators involved in the radiolabelling are still to be addressed.

## Figures and Tables

**Table 1:** Radioactive residues in different parts of the disposable cassette (ML Eazy) gathered based on precursor (columns A and B) or on radionuclide (columns C and D) used. Numbers are given as percentage of the starting radionuclide activity (means, *n* = 12). ^*∗*^Values not corrected for geometry.

	DOTATOC	PSMA-617	Yttrium-90	Lutetium-177
Reactor	3.5	2.3	4.8	1.1
Delivery vial	12.8	13.8	12.6	13.2
Tubing^*∗*^	0.3	0.0	0.0	0.8
CM cartridge^*∗*^	6.7	0.1	7.9	3.6
Sterilizing Filter^*∗*^	0.6	0.1	0.8	0.3

**Table 2:** Evaluation of radiochemical purity of ^90^Y- and ^177^Lu-DOTATOC preparations performed by a manual, semiautomated (ADD-2), and automated (ML Eazy) approach.

	^90^Y-DOTATOC	^177^Lu-DOTATOC
*Manual syntheses*		
Syntheses failed	0/77	0/43
Uncompliant preparations (RCP < 97%)	6/77	3/43
*TLC*		
Free-radionuclide (%)	0.2	0.2
Hydrolysed products (%)	0.0	0.1
Labelled product (%)	**99.8**	**99.7**
*HPLC*		
Free radionuclide + hydrolysed products	1.2	0.8
By-products	0.5	0.5
Labelled product	**98.3**	**98.7**

*Semiautomated syntheses*		
Syntheses failed	1/63	0/88
Uncompliant preparations (RCP < 97%)	1/62	0/88
*TLC*		
Free-radionuclide	0.23	0.25
Hydrolysed products	0.27	0.15
Labelled product	**99.5**	**99.6**
*HPLC*		
Free radionuclide + hydrolysed products	21.3	1.0
By-products	0.4	0.2
Labelled product	**98.3**	**98.8**

*Automated syntheses*		
Syntheses failed	1/24	1/26
Uncompliant preparations (RCP < 97%)	1/23	0/25
*TLC*		
Free-radionuclide	0.2	0.3
Hydrolysed products	0.3	0.4
Labelled product	**99.5**	**99.3**
*HPLC*		
Free radionuclide + Hydrolysed products	1.9	0.7
By-products	0.4	0.9
Labelled product	**97.7**	**98.4**

**Table 3:** Evaluation of radiochemical purity of ^90^Y- and ^177^Lu-PSMA-617 preparations performed by the automated automatic synthesizer ML Eazy.

	^90^Y-PSMA-617	^177^Lu-PSMA-617
Syntheses failed	0/5	0/5
Uncompliant preparations (RCP < 97%)	0/5	0/5
*TLC*		
Free-radionuclide	0.2	0.3
Hydrolysed products	0.1	0.1
Labelled product	**99.7**	**99.6**
*HPLC*		
Free radionuclide + hydrolysed products	0.5	0.3
By-products	1.0	0.2
Labelled product	**98.5**	**99.5**
